# The Hospital. Nursing Section

**Published:** 1903-01-17

**Authors:** 


### The Hospital.

### Nursing Section.

Contributions for this Section of "The Hospital" should be addressed to the Editor, "The Hospital" Nursing Section, 28 & 29 Southampton Street, Strand, London, W.C.

No. 851.—Vol. XXXIII. *SATURDAY, JANUARY* 17, 1903.

### Notes on News from the Nursing World.

### THE QUEEN'S MILITARY NURSING SERVICE.

The temporary offices of the Queen's Military Nursing Service, in Whitehall, have now been vacated, and accommodation for the whole department has been found at 68 Victoria Street. The offices of the matron-in-chief consist of a suite of rooms on the second storey from the top; they are bright and cheerful, and as all but one face the south they have the advantage of the morning sun. They consist of the matron's office and those of her assistants, including the principal matron for home service. A large room behind is set apart for six clerks. The small room occupied by the lady typist serves as a waiting-room for candidates. All the offices are papered with green striped wall-paper, surmounting a brown dado, and they are, of course, simply furnished. On a lower floor is the large board-room. In consequence of the publication in The Hospital of the new regulations for the Queen's Military Nursing Service, a large number of applications from nurses have been, and are still being, received at the offices.

### A NEW DEPARTURE IN ARMY NURSING.

The opening by her Majesty the Queen of the nurses' new quarters at the Herbert Hospital, Woolwich, marks the first important innovation in the military nursing system, although the nurses, who are now being appointed, are not, as was at first inferred, to take the place of the orderlies. The staff will consist of the matron, seven sisters, twelve staff nurses, and about twenty-five orderlies, and the training of the orderlies will be more thorough in the future than it has hitherto been. With regard to the training of the nurses, it will not now be necessary for every nurse who is accepted for military appointment to go through a preliminary course at Netley. The three years' course in medical and surgical nursing at a civil hospital recognised by the Advisory Board will be sufficient, and the specialised military part of the training will be acquired in the hospital to which the nurse is attached after her admission to the service. The new quarters at Woolwich are expected to be ready shortly, and they will be opened as soon as a convenient date can be fixed.

### HONOUR TO A LADY SUPERINTENDENT IN IRELAND.

The Rotunda Hospital, Dublin, has had presented to it a portrait, life-size, of Miss Sarah E. Hampson, its first lady superintendent. The presentation is from Dr. W. J. Smyly, and it is stated that when he made the proposal to the Governors his generous offer was received with enthusiasm. The picture of Miss Hampson is to hang in the nurses' dining-room in a similar position to that of the founder of the hospital, Dr. Mosse, in the board room. The action of Dr. Smyly illustrates the good feeling prevailing between the medical and nursing staffs in most hospitals, and the esteem in which the latter are held by the former, who are quick to see, and ready to admit, the futility of their labours when their instructions are set aside or are not carefully carried out.

### THE WILL FOR THE DEED.

A wide movement was started for making a presentation to Miss Wilson, Miss Paget, and Miss Fynes-Clinton in recognition of their persevering service in the cause of the Midwives Act. But knowledge of the intention having come to the ears of the ladies themselves, they declined to receive any present and preferred to take the will for the deed. We are almost sorry in this case that the midwives were not able to give public expression to their gratitude, but we recognise how often presentations are obligatory, and admire the self-denying ordinance of the three ladies.

### THE COTTON-WOOL FOLLY.

Last week we mentioned the case of a nurse who was injured by a piece of hot ash from a cracker flying into her eye. This was an accident that could not have been anticipated, but we hear of another of which the same cannot be affirmed. A nurse dressed herself as Father Christmas, her costume being covered with cotton-wool, which took fire, and before assistance could be rendered, she was burnt in the face. Being a member of the Royal National Pension Fund, she has obtained sick pay, but we doubt whether anyone who undertakes such obvious risks ought to be entitled to it. Over and over again the folly of using cotton-wool for decorative or costume purposes, except when, by treatment, it has been rendered uninflammable, has been pointed out; and people who will persist in running into danger could not expect much pity if they suffered in pocket as well as in body.

### THE AMERICAN SUPERINTENDENT.

At the recent annual conference of the Association of Hospital Superintendents, held at Philadelphia, U.S.A., the question whether the superintendent of the training school should be also matron of the hospital, was an animated topic of debate. Miss McKechnie, superintendent of the Hospital for Women and Children in New York, contended that in a large hospital it was impossible for one woman to combine the duties. Dr. Rowe, medical superintendent of the Boston City Hospital, at once disposed of this very sweeping statement by the effective rejoinder that at his own and several other large hospitals the leading woman official is superintendent of nurses and matron. He rightly added that much depends upon how the matter is organised. In the leading English hospitals the plan of combining in one person the obligations of matron and superintendent of nurses unquestionably answers well. It is when there is a divided authority, as in the case of workhouse infirmaries, that friction ensues. Whether the lady invested with supreme authority in respect to the training school and the housekeeping arrangements is called superintendent or matron is of comparatively little consequence. If she requires assistance it is always easy to give her that of an assistant matron or book-keeper. But whatever her assistants are called, she should be supreme.

### PAUPER ATTENDANTS

Mr Wethered in his report on Poor Law Administration in 1901 and 1902 dwells with satisfaction upon the fact that since 1899 the number of pauper attendants in the sick wards in his district has been reduced from 312 to 222. He thinks that some Boards of Guardians fully realise that if paupers are capable of discharging the duties of ward assistants they are generally also capable of earning their living outside the workhouse, and "rightly object to offering any inducements to people to remain paupers on the ground that it is cheaper than paying assistants in the sick wards." The tabular return appended to the report shows that on January 1st, 1899, there were 132 nurses on day duty and 43 on night duty, whereas on January 1st, 1902, they had increased respectively to 153 and 57. The present average is one nurse to fifteen patients.

### AN ARMY SISTER AS LECTURER.

Miss Brereton, whose name will be familiar to our readers, has made a very successful appearance as a lecturer. At Cromer, where she gave an exhaustive description of her work among the sick and wounded in South Africa, illustrated by a series of limelight views, she had an enthusiastic reception the other night. In replying to a vote of thanks, which was moved by Mr. Broadhurst, M.P., Miss Brereton said she felt that everyone had an immense responsibility in this great Empire, and that if she could teach any that they had a duty to perform in the work of the nation her labours were justified. We are glad that a nursing sister, whose brilliant services during the war secured her the Royal Red Cross, should be able to turn her experiences to such excellent account.

### PRIVATE NURSES FOR MENTAL CASES.

The committee of the Retreat, at York—a registered hospital for the treatment of mental diseases—which has just issued its 106th annual report, have arranged to provide thoroughly experienced nurses for the nursing of patients in their own homes or privately. The nurses will have had at least three years' training at the Retreat. It may be interesting to add that the training is of a very complete character, and quite up to date. Each approved candidate is required to engage to stay for four years, during two of which she is a probationer, the third year a staff nurse, and the fourth year if opportunity arises, and she is found suitable, she serves on the private staff. There is a course of lectures each winter service during the first two years by the medical officers dealing with outlines of anatomy and physiology, first aid to injured, elementary explanation of the principal forms of disease, nursing the sick and hygiene, causes and varieties of mental diseases, and the treatment of mental disorders. Demonstrations in bandaging, bed-making, and practical nursing are given. In the third year the lectures on mental and nervous diseases are continued, and there is further instruction in the anatomy of the muscular and nervous systems. A class in Swedish medical gymnastics is held ; a course of instruction in massage is given, and finally there is a class on invalid cooking. An examination takes place at least once a year, and at the end of the second year each nurse is expected to enter for the certificate of proficiency in mental nursing given by the Medico-Psychological Association of Great Britain and Ireland. At the end of the fourth year the special Retreat certificate is awarded, and each nurse who receives it is also eligible for the "William Tuke" medal at the discretion of the committee.

### THE UNTRAINED NURSE AT DAVENTRY.

The Daventry Board of Guardians last week decided to dismiss a nurse who was accused of neglecting the patients in the workhouse infirmary ; and the evidence of the medical officer in the matter affords a justification for the action of the majority. But far more important than the personal question is the issue raised by Dr. O'Rafferty on the general question. He frankly told the Guardians that the nursing in Daventry Workhouse Infirmary is "a farce," and asked whether any members of the board, if ill, "would want to be nursed by an ex-cook or an ex Salvation Army lass?" In short, he insisted upon the necessity of substituting a trained for an untrained nurse. The force of his argument was recognised by the chairman, who said that "he himself had held the same opinion for years and years." But why, then, has nothing been done, especially as the chairman subsequently stated that the medical officer had often individually complained to the Guardians of the wretched nursing ? Even now it does not appear certain that a trained nurse is to be engaged. The failure of their duty to the patients by the guardians seems to call for more severe condemnation than the neglect of the nurse whom they have dismissed.

### THE PERSEHOUSE PENSION.

The correspondent who asked for information respecting the Persehouse Pension, and probably other readers, will be interested to learn that this charity was founded by Mr. Henry Persehouse Parkes, of Birmingham, who died on May 17th, 1901, and left £50,000 or thereabouts for the purpose. The object of the Persehouse Pension Fund is to assist, by way of pensions, or otherwise, aged or distressed persons, male or female, belonging to the upper or middle classes of society. They must be either natives of the counties of Stafford or Worcester, or for ten years or upwards have resided in one or both of them, and they must also have been reduced to poverty by misfortune and not by their own improvidence or neglect. The trustees have established pensions not exceeding £50 each, which, in the first instance, are granted for a year, and renewed annually at their discretion. Persons who desire to apply for pensions can obtain forms from the solicitors for the trustees, Messrs. Pensent and Co., 6 Bennett's Hill, Birmingham, to whom also two written testimonials have to be sent. But we understand that the trustees have allotted all the funds at present available for pensions, and that therefore any applications which are received will have to stand over until further funds are at their command.

### THE ROYAL HOSPITAL FOR CHILDREN AND WOMEN.

On account of the limited accommodation in Stamford Street, it was impossible to hold the usual Children's Entertainment at the Royal Hospital for Children and Women, Waterloo Bridge Road. There was, however, a tree for the children on January 6th, and on the 8th and 9th subscribers were invited to see that the work is being carried on as well as possible in such restricted quarters. The old hospital is being rapidly pulled down, and the new building, according to contract, will be ready in eighteen months' time. It will cost £30,949, and will form a handsome corner block. The nurses' home will occupy the position of the house in which the patients are at present housed, and will be part of the block.

### NURSES' ENTERTAINMENT AT YORK COUNTY HOSPITAL.

The nurses' entertainment at York County Hospital in the early days of January is a special and enjoyable feature. If peals of laughter and the heartiest of applause are any criterion, the traditions of the institution were maintained this year by the present staff. The varied programme included several part songs, a diverting sketch, and a recitation, followed by the appearance of a nigger troupe. The droll humour of the senior nurse, who conducted the troupe through their solos and choruses, caused much merriment amongst the audience. That in a few hours these little niggers with their laughing black faces and gaudy costumes would be stiff and prim in neat uniform was hard to realise. All throughout the festive season worked with hearty good will to give the inmates of the wards pleasure and make them forget for a time their aches and pains, but of all the many little plans carried out for their amusement the patients declared that "th' nusses' concert was t' best o't lot."

### ROYAL BRITISH NURSES' ASSOCIATION.

The next sessional lecture under the auspices of the British Nurses' Association will be given at 10 Orchard Street, W., on Thursday, January 22nd, at 5.30 P.M. The lecturer will be Dr. Robert Jones, medical superintendent of Claybury Asylum, and his subject will be "Dipsomania, its Causes, and Cure."

### DEATH OF A CORK NURSE FROM TYPHUS FEVER.

The epidemic of typhus fever which began at Cahirciveen last November has subsided, but among the victims has, unfortunately, been one of the members of the nursing staff attached to the South Charitable Infirmary and County Hospital at Cork. Miss Anna Fitzgerald, the nurse in question, had temporary charge of the Fever Hospital at Cahirciveen, the permanent matron, as well as one of the Sisters of Mercy, being prostrated with typhus, and it was not until the disease had almost run its course that she herself contracted it. Her death, at an early age, has provoked widespread expressions of sympathy, and at a meeting of the house committee of the South Charitable Infirmary, a resolution of condolence with her relatives was passed. Beautiful wreaths were sent by the matron and nursing staff at the funeral.

### THE CHELTENHAM DISTRICT NURSES.

The contribution of the Cheltenham Guardians to the District Nursing Association is larger than we intimated on January 3rd. On condition that the district nurses undertake the charge of pauper cases in the country parishes of the union as well as the nursing in the town, the Guardians increased the amount of their grant from £70 to £170 a year. But the work of the two members of the staff in the country parishes is not, we understand, by any means confined to nursing pauper cases, which are not very numerous. They nurse amongst the labouring classes generally whenever they are wanted, charging a small fee for maternity cases, and nothing at all for general cases. This is as it should be.

### PING-PONG AND THE SOUTHGATE NURSE FUND.

The feature of the seventh annual report of the Southgate Village Nurse Fund is that it records the contribution of £56 as the result of a ping-pong tournament. There are some who fail to understand the charm of the game, but so long as it is attractive enough to excite such substantial interest as this amount denotes, it can be turned to a very useful account. The Southgate Fund also benefited by a church parade instituted by the various Friendly Societies, and by a surplus from the Coronation festivities. The nurse paid nearly 4,000 visits during the year, and received, after the ping-pong match, a handsome gold watch as a mark of the esteem in which she is held for her services among the poor.

### A NEW NURSES' ORGANISATION.

Under the auspices of Mrs. E. Palmer, who was trained at the Royal Free Hospital, Gray's Inn Road, as superintendent, a number of nurses have formed themselves into an organisation with a residential home. It is called the Colosseum Nurses' Association, and every member is required to hold a three years' hospital certificate. Each nurse is to receive the whole of her fees, less 5 per cent., which is to cover office and incidental expenses. The names of the patrons of the Association include some of the best known medical men in London.

### SHORT ITEMS.

Convalescent home letters are urgently needed by the Ladies' Association of the Tottenham Hospital and will be gratefully acknowledged if sent to the Hon. Secretary, Ladies' Association, Tottenham Hospital, N.—The s.s. *Nubia*, which arrived at Southampton on Sunday, had on board Sisters C. G. Keel, M. B. Bennett, L. Badgee, E. M. Hodgson, A. S. Dawney, K. Hodkins, and W. Potter.—Miss Ethel M'Call, Lady Howe, and Miss Violet Brooke-Hunt have joined the Committee of the Union Jack Club, the new central institute for sailors and soldiers.

### The Nursing Outlook.

"From magnanimity, all fear above;
From nobler recompense, above applause;
Which owes to man's short outlook all its charm."

### POOR LAW INFIRMARY TRAINING SCHOOLS.

The proposal made by the departmental committee on workhouse nursing to manufacture for Poor Law purposes, and incidentally to throw upon the market for employment by the public a large number of one-year trained "nurses," draws attention very seriously to the position of the workhouse infirmaries as places in which to obtain a training, and as a rider to this to the further question of the position which will in future be held by nurses of all degrees who obtain their certificates from Poor Law training schools.

Up to the year 1898 the whole subject was in a condition of chaos, the indefiniteness which hung over the meaning of the word "trained" leading to many complications. For many years the Workhouse Infirmary Nursing Association did a great work in filling the vacancies, while the establishment of admirable training schools at some of the great Poor Law infirmaries helped to relieve the pressure, and with patience—if only the Guardians at large had but recognised what was being seen by the more enlightened among them, namely that the conditions of the service must be improved if its attractiveness was to be increased, and if the Local Government Board had but taken up a strong position in the matter—the problem might have been solved by the ordinary law of supply and demand.

Nothing could be more preposterous, however, than the position of affairs which arose—a position which threatened again to debase the nursing in workhouses; for all the world began to train nurses and to give certificates. Fortunately the situation was for the time saved by the Nursing Order of 1898, which, little as it did to compel the guardians to provide proper nursing for the sick in their charge, did at least lay down a definition of what was meant by training, and did at least show to such of the workhouse probationers as would listen to words of warning (which we have persistently given), that unless the school they selected was one recognised by the Local Government Board, they would find themselves debarred from all chance of attaining to any of the higher posts in the Poor Law service. The essence of this Order lay in the definition of the character of the training schools which would alone be recognised, and in the demand for a three years' course at such a school. The promulgation of this Order was naturally regarded by nurses at large as a matter of much importance, since it, at last, gave official sanction to that more prolonged course of training which had long been maintained by the matrons of the leading schools to be essential.

Thus out of chaos order seemed to be arising, and the trained nurse seemed to be emerging from her trials with a defined position. If only the Local Government Board, having gone so far, would but have gone one step further and issued a strong order, somewhat on the lines of that now in operation in Ireland, compelling all Guardians to accept the standard already loyally accepted by the better of them, the effect would have been not only to give to the sick poor in our workhouses—the worn out failures of our social system—the kind of nursing which they certainly deserve, but also to squelch the smaller and more inefficient so-called training schools, and so to protect the public from being imposed upon by elaborate but meaningless certificates. Instead of doing this, however, the Departmental Committee have now recommended the re-establishment, on a firmer basis than ever, of the one-year-trained nurse who, although only licensed, if we may so term it, to attend paupers under supervision, is to be allowed to go out and earn her living by nursing the public on her own responsibility. We have already pointed out how disastrous must be the effect of this course upon the public and upon the nursing profession, but the question is in our opinion well worthy of the most serious official consideration whether this recommendation, if carried into effect, may not add new difficulties to those already existing in the way of obtaining good nursing in workhouses.

There is much reason to fear that in the end all nurses trained under the Poor Law will come to be tarred with the same brush in the public eye. The public, certainly, will never take the trouble to draw any distinction between the higher and the lower training. People will be content with asking whether a nurse is "hospital trained" or "workhouse trained" and all those who are "workhouse trained" will be lumped together. Thus it will happen that the wide gulf between these two groups of nurses, which the Order of 1898 tended so much to remove, will once again come into view much to the detriment of all who are associated with the Poor Law. The result must be to deter the more ambitious among the well-educated probationers from entering the Poor Law service. Nurses hold firmly to their professional position, they are as a class pervaded by a strong *esprit de corps*, and it is by no means improbable that a feeling may grow up among them leading them to regard the work of training these one-year-trained blacklegs as something unprofessional, derogatory to their position, and disloyal to their colleagues; and if such a feeling should take root, as it might well do should these untrained but "qualified" nurses increase in number and in hunger, we might find a taboo set up against the Poor Law service which it would take many years to remove.

### Lectures to Nurses on Clinical Chemistry.

By Carstairs Douglas, M.D., B.Sc., F.R.S Edin., Professor of Medical Jurisprudence and Hygiene, Anderson's College Medical School, and Dispensary Physician, Samaritan Hospital, Glasgow.

### LECTURE IV.

Tests for Sugar in Urine (*Concluded from page* 194).

Another simple and useful test is that named after Trommer. The chemicals required are solutions of sulphate of copper, and of caustic potash (liquor potassæ), which are to be found in all test-rooms. To perform the test, one takes about an inch of urine in a test-tube, adds a few drops of the blue copper sulphate solution, and then the liquor potassæ carefully, drop by drop, till the light blue precipitate which forms at first is all redissolved. The tube should be shaken all the time. If the upper part of the column of liquid be now boiled, the presence of sugar will be detected by the clear blue fluid becoming first turbid, and finally opaque and yellowish red. The copper compound here again, as in Fehling's test, undergoes what is called "reduction" from one state to another (it is really robbed of some oxygen), and this is accomplished by the sugar.

These tests are very simple, and we would not need to employ any others were it not for the fact that certain other substances may occur in urine which also, like sugar, have the power of "reducing" copper salts to this yellowish-red form. I may say, however, from the experience of many hundreds (if not thousands) of urinary analyses, that these substances practically never produce that distinctive and marked effect with either Fehling's or Trommer's test which sugar, to the amount of 1 per cent., or, say, 4½ gr. per ounce would. But when the sugar is scanty, it is impossible to say whether the slight and equivocal reduction it effects is really due to it, or is occasioned by some other body, e.g. urates. So that we are obliged sometimes to use another test with regard to which no fallacy can arise.

(3) *The Yeast or Fermentation Test.*—On this we may fall back when we are in real doubt. You are all doubtless aware that yeast, which consists of a cell-like mould, has the power of producing a peculiar effect on solutions containing certain sugars, whereby gas is developed and the mixture seethes and froths. The seething is due to the production of carbonic acid gas, which, along with alcohol, is produced during the fermentation. It is for this purpose that yeast is used in baking; it causes fermentation with sugar in the moist dough, and the bubbles of gas forcing their way through the mass cause it to swell up and become light, porous, and aerated. Now, though grape sugar can and does ferment readily, other substances in urine like uric acid or urates that may mislead one in testing, do not. So if doubt remains, let a test-tube be filled with urine, a small piece of yeast added, and the whole inverted over a little dish containing mercury. The tube must be held with its mouth under the mercury by means of some suitable clamp. Tube and dish may now be placed on the mantel-piece or near the fire, where, in the presence of very small amounts of grape sugar (*e.g.* 1–500) bubbles of gas will collect after the lapse of 24 hours in the upper (closed) end of the test-tube. The tube is placed over mercury, because if water be used it can dissolve the gas produced so that it could thus escape observation. Before we leave the subject it may be stated that if, before testing for sugar, the urine is found to be albuminous, the albumin must first of all be removed. To accomplish this boil the urine, accidulate with acetic acid, and filter through a little filter paper. Some of the clear fluid which drops through may then be used for Trommer's or Fehling's test.

*Tests for Blood.*—There is only one chemical test for blood which a nurse makes use of, and that is what is called the guaiac test or reaction. Guaiac is a resin obtained from the lignum vitæ tree of Jamaica, and is soluble in spirit, forming a reddish-brown fluid with a pleasant smell—tincture of guaiac—one of our reagents. The other is a solution of peroxide of hydrogen (well-known to ladies on account of its power of bleaching the hair) in ether, and named ozonic ether. The test depends on the fact that guaiac when oxidised, *i.e.*, when it is caused to combine with oxygen, gives a blue colour. Now, though peroxide of hydrogen (in the ozonic ether) can yield oxygen, the guaiac cannot seize it directly, it needs an intermediary. For this latter post blood is admirably suited, and by virtue of its colouring substance (hæmoglobin) can take the oxygen from the peroxide and hand it on to the guaiac, producing a blue tint. To perform the test, fill a test-tube with some of the suspected urine to the height of one inch, taking a little of the sediment if any be found, since this often contains blood cells. Now add two drops of the tincture of guaiac (which should be fresh), when it will be noticed a whitish-brown opacity is caused. This may be entirely disregarded, as it is due to the resin being precipitated from the spirit by the watery urine, in which it is insoluble. We therefore pay no heed to it, but add ozonic ether, in amount equal to half that of the urine, and shake very gently. The light ethereal solution quickly separates out and rising, floats on the top, and very soon, if blood is present, a delicate blue ring is seen (varying in depth and intensity according to the amount) where the ether and the opaque urine join. It is the formation of this blue zone which betokens the presence of blood. This test is not infallible, as pus and certain other substances containing animal or vegetable cells (which are active oxidisers) may also strike a blue colour with guaiac. But it is sufficiently delicate for most clinical purposes, and it is the test a nurse should know. Sometimes instead of ozonic ether, old (well-oxidised) oil of turpentine is used.

*Bile in Urine.*—It is often important to be able to say whether or not a urine contains bile, and one may hazard a guess even from the colour, which is usually a brown with a shade of green in it. Patients who have been taking carbolic acid may also pass a urine of dark tint, but this can be distinguished from bilious urine on shaking, for the froth in the latter case is tinged yellow, while in carbolic urine it remains white. (1) The iodine test. If on the top of some bilious urine in a test-tube a little tincture of iodine diluted with ten times its volume of rectified spirit, be poured, the presence of bile is indicated by a fine green band where the lighter alcoholic liquid floats on the heavier urine. If not distinct at first, the tube may be gently shaken. The green band is often very clear and beautiful. (2) The Gmelin test. The reaction, named after the chemist who introduced it, consists in noting the effect produced on bilious urine by nitric acid containing a little nitrous acid. To perform it, place a thin film of the urine on a white porcelain slab, and allow a drop or two of the acid to fall in the middle of it. If bile is present, a play of colours is seen in the form of concentric rings, showing green, red and blue. Or a little of the bilious urine may be run through filter paper, and on the yellow-stained paper drops of acid allowed to fall in the same way.

### "The Hospital" Convalescent Fund.

The Hon. Sec. begs to acknowledge with thanks the receipt of 2s. 6d. from "E. C. N."

### Nursing in Smyrna.

BY A CORRESPONDENT.

IT was a close, sultry evening when I went to my first case in Smyrna—one of typhoid fever. It was in a Greek family and my arrival was decidedly trying. I was ushered into the drawing-room, where all available relatives were collected to inspect the "English-trained nurse"—a novelty in those days; the hostess greeted me, and then solemnly introduced me to everyone in the room in turns; then came a pause, in which they all regarded me steadily. I suggested that it might be well if I were introduced to my patient, whom I heard was very ill; thereupon I was escorted upstairs by a contingent of the company, and after putting on my uniform was taken to the sick-room.

### Keeping the Patient Undisturbed.

The room was furnished with as many curtains, rugs valances, etc., as it was possible to cram in, the window was tightly shut, and, there being no fireplace in the room, the atmosphere can better be imagined than described. The principal idea is to keep the patient undisturbed, and well wrapped up, and to exclude every possible breath of fresh air. My patient had been ill about four or five days, and had evidently not been disturbed. She was tossing about, with a temperature of 105°, in a large bed, with a soft flock mattress; the under sheet was beautifully rolled into a hard rope under her back, the top sheet being in a ball at the foot of the bed, the patient being covered by blankets and a lace quilt; the temperature of the room was 100°. Every available corner was taken up with plates and cups containing the remains of delicacies which the too kind relatives had offered to the poor sufferer to tempt her appetite.

### A Revelation.

I first made a clean sweep of all these things and to their amazement told them I should only require fresh milk for the present. Then I started my preparations for sponging and settling the patient; this caused their eyes to start with horror, as the idea of washing a sick person and disturbing her to make the bed evidently seemed little short of murder in their eyes. My patient was very ill indeed and would need all that careful nursing could do to pull her through, and as my experience of these hot climates is that fevers take much more rapid turns for the worse than in England, I knew she would need constant feeding and watching. Of course I stayed with her that night during which she became violently delirious; besides having to constantly watch her to prevent her getting out of bed, the mosquitoes had to be kept away by continual fanning. They collect round a sick bed and take special pleasure in collecting round the poor nurse's ankles, knowing that she can't drive them away, her hands being otherwise occupied. I have since discovered that sprinkling a little turpentine on the stockings will keep them away.

### The Greek Nurse.

I stayed on duty till the following evening, when the doctor had arranged for a Greek night nurse to relieve me. The patient's relations could not understand why a trained nurse should need "off-duty" time or sleep, their idea of training evidently being that she underwent some process by which she could dispense with both. The night nurse duly arrived but did not impress me favourably, she was very large and very fat, and I could better imagine her sleeping than watching a patient. I left the minutest instructions as to the feeding, etc., and went to bed very tired but not easy in my mind. I woke up again a couple of hours later, and listened if there was any sound, my room being next to the patient's. At first I heard nothing but the patient muttering, then I distinctly heard a snore, which brought me out of bed instantly, as I knew that did not come from the patient. I flew into the room and found what I expected, the nurse fast asleep in an armchair by the bed, and the patient sitting up in the act of getting out of bed. I called to the nurse who slept on peacefully, hearing nothing. Accordingly, after getting the patient back into bed, I shook the nurse, none too gently, I am sure, and asked her what she meant by going to sleep. She swore that she was not asleep, only closing her eyes; but, of course, I had to remain with the patient for the rest of the night. Next day I told the doctor, who said he was afraid they were all more or less alike; however, after trying two or three more, we got one who did fairly well, and had some intelligence.

### The Difficulty with the Relations.

The relations were extremely kind and well meaning, the difficulty being to keep them out of the sick-room as much as possible, without hurting their feelings. Their idea of tempting the patients' appetite was firmly fixed in their minds, and I knew that if I relaxed guard over my patient, she would be fed with chocolate creams, or some dainty she used to like. However, notwithstanding all difficulties, she pulled through; but the convalescence was the time to call forth all one's tact, firmness, and good temper. All nurses know what a convalescent typhoid's appetite is, and when it was a case of the patient crying for food on the one hand and the friends trying every ruse on the other to give her that food, as they were sure she needed feeding up, it was the most trying part of the illness. I found the doctors most courteous and ready to uphold the nurse's authority, and very appreciative of having someone whom they could trust to follow their instructions *au pied de la lettre*, and not with variations at the patient's desire.

### Turkish Objection to the Telephone.

A nurse has to be ready in case of emergency, as very sudden development of dangerous symptoms take place, and the doctor cannot be found quickly, there being no telephonic communication at all—the Turks, imagining the telephone is a means used for communicating with the Evil One, forbid its use. The English community, of course, is more up to date, and would probably make things easier for the nurse; but a private nurse's work is principally among the Greeks, Armenians, French, and possibly even Turkish women in harems. The doctors, who are mostly Greeks and Armenians, speak French, and a few speak English; but the patients mostly speak Greek and a little French, rarely English. The servants are all Greeks and Armenians, and only speak their own language, which is very bewildering to a new comer.

### Lodgings and Expenses.

Nurses must be very careful where they lodge, as the accommodation is very bad for anyone starting on her own account. Food is cheap, but then living and clothing are very expensive. A nurse's great difficulty is getting suitable food for convalescent patients, such things as milk puddings, custards, etc., being almost unknown amongst foreigners, and very often the nurse has to make them herself, in kitchens not at all suitable for the purpose. The water supply also is very bad, and the sanitary arrangements are most imperfect, rendering disinfection difficult and unsatisfactory.

### Words of Advice to Nurses.

BY ESTHER H YOUNG.

### LECTURE IV.

In our last lecture we thought about carefulness, that, as we are Nurses, there is special care needed. Your other lecturers teach you how to tend and care for your patients; may I now speak to you more particularly about yourselves?

It is quite impossible for us, in considering points connected with our work or our persons, to entirely leave out the religious side of the question, and though I must not inflict on you too much "Sunday teaching" nor urge you to do this, that or the other religious duty, yet—as your fellow Christian, as your fellow nurse, as your fellow woman—for your own sakes and for your patients' sakes, I would affectionately ask you to try to be careful about your religious duties. I know something of your temptations. I know something of your dangers; so may I offer a few simple hints, even though it is not my "business" to advise you!

I would ask you to be on your guard against the temptation to give up first one good practice, then another. At home, on Sunday, you always attended public worship with your family. In hospital, when Sunday comes, you are free to do as you like, and you are tired, so you take a "yellow-back" and lie on your bed, or sit by the fire or out-of-doors. How grieved your mother would be at this neglect of duty (you know that joining in public worship is a duty!) It is fatally easy to slip into these careless habits. But fight against them, new "pro." or tired third-year nurse! From your babyhood you have said your prayers night and morning in your bedroom, and at home you never thought of breaking through this habit. There comes a day in this hurried life when fear of that late mark sends you down to breakfast, having for the first time in your life omitted this duty. Do let it be the last. Keen as all nurses should be about punctuality, it would be better to be late than early, if "early" means that you have omitted your prayers. But the sooner you learn to get up at once when called, the easier will you find it to do all you should before leaving your room. Then perhaps there comes a night when you are tired out and cross, and everything has "all gone wrong together"—nurse has been snappy and sister does not understand; you get into hot water when somebody else was to blame, and (how often) you are inclined to think that it's "no use trying!" You say, "I feel too tired to-night to bother about prayers. And so for the first time in your life, you, poor weary child, neglect to take all these troubles and failures to the loving Father Who is waiting to help you.

With all affectionate tenderness I would say, don't ever give up your daily prayers—no, nor let Chapel prayers do instead. "Talking goody" or any religious cant is very distasteful, and there is a holy reserve which would keep us from bringing in sacred things at unreasonable times—but I do think we are sometimes apt to err a little bit on the other side. When we see another in danger of drowning we should not let her drown, should we?

Let us surely in this matter, as in others, help one another in any way we can, not in any set form of words or special phrases, but just as opportunity offers—kindly, gently, cheerfully, and wisely if possible. We shall do less, much less, by words than by example. Let us be ourselves, just simple, childlike, faithful Christians, remembering that we are members of Christ and members one of another.

I think that we nurses are so anxious to take care of other people's bodies, that we are apt to forget the cultivation of their minds and of our own. Then people say of us that we are narrow-minded, we think and speak only of shop. Perhaps you will say "we have so little time for cultivation of mental capacities, education of mind." But still we have time—and how do we use that time? Do we ever read? And if so, what? Always a novel, nurse? And always the latest, good or bad? Some indeed are very bad, quite unfit for any woman to read, and as bad for the health of their minds as smoking would be for their bodies. If we are distinctly told a book is bad—someone has seen it reviewed, and warns us about it—why must we get it to see what it is like? We don't drink poison to see what it is like—then don't let us read bad books, no, nor even foolish or frivolous ones. If for no other reason, for the good of our patients let us educate our minds. Many of you go into hospital interested in some particular art or science—don't drop it altogether, keep up your drawing, your music, your needlework, etc., etc. It is true you cannot leave your work and put the ward to inconvenience because you wished to study, you must "give up" many things for your patients' sakes, and you have come to hospital to learn nursing, but you need not entirely give up your particular hobby.

Probably you have a good library and the daily papers are sent to your rooms—read them and be "up" in the topics of the day. Besides being better for yourselves it will be infinitely better for your weary sick folk, if you will take an interest in such subjects.

Further, I would beg of you to keep up your outside ties—and first, your home ties. In these days of organised recreation for nurses there is a danger lest relations and friends become "dropped." Never neglect your home letters. I have known a nurse who for ten years (with only two or three exceptions) has written to her mother every week regularly. Make a definite plan about your letter writing to father or mother or little sisters or brothers; write to your old schoolfellows, your girl friends. If you think your work will not interest them then that is an excellent reason for keeping up the education of your mind by reading wholesome books, and by keeping in touch with daily events outside the hospital. Go home, if home is near enough—go to your friends. Go out at least every other day. If you are in London you live in one of the most famous cities of the world; learn something about it. Go to St. Paul's, Westminster Abbey, the Tower, the Mint, and any other place of interest which may be near to you. Find out all the "Dickens" bits—many of them may be within your reach—and sometimes look in the shop windows for there is much there to amuse you.

Or if you train in a country hospital, surely one need hardly mention in detail the various beauties to be found there to interest you, the wonderful and gradual growth of nature during the various seasons of the year, the different songs of the birds. Years ago the Vicar at home used to tell us that he always heard the birds singing their hymn of praise to God as he walked up the hill in the early morning to church. Watch the growth of the trees, and if you can cultivate a few flowers, or take a real interest in those belonging to friends.

Speaking of going out brings one, of course, to the subject of dress. I mean walking dress. Outdoor uniform is perhaps provided for you, if to wear it is according to rule. In these days, unfortunately, the wearing of hospital uniform does not necessarily mean that the wearer is a nurse, or even a moral woman—women who have had no training at all wear uniform. To my mind, such a custom is, of course, ridiculous and not exactly right, for the wearer appears to the world to be what she is not; but as it is done it is just as well that nurses should be free to choose in this matter. In these days, too, of cycling and tennis, it is a good thing that nurses should be allowed to wear ordinary walking dress.

*(To be continued.)*

### Everybody's Opinion.

[Correspondence on all subjects is invited, but we cannot in any way be responsible for the opinions expressed by our correspondents. No communication can be entertained if the name and address of the correspondent are not given as a guarantee of good faith, but not necessarily for publication. All correspondents should write on one side of the paper only.]

### THE JUNIUS S. MORGAN BENEVOLENT FUND.

"R. N. P. F., No. 1217," writes: Seeing such a splendid account of the Royal National Pension Fund for Nurses during 1902, I would like to send my thanks to all connected with it for the great boon it has been to me. Retired from work I have all I need in a comfortable pension, and before receiving it, during the time of ill health, I had substantial help from the sick pay. Happily I have never needed to apply to the Benevolent Fund, but surely every nurse should feel it a duty and pleasure to subscribe to it for the sake of less fortunate sisters. The poorest nurse could spare 1s. a year, and yet how little was received for it during last year.

### THE NURSING OUTLOOK.

"J. H." writes: I for one am glad that you are to devote a page of your valuable paper to nursing questions of the day. You invite suggestions, so may I mention a few points, which constantly seem to come up?—(1) The inequality of a three years' certificate. One nurse has to pass an examination, or may be more than one to gain her certificate, being examined by one of the staff, or better, as here, by an outsider. Another gets hers, in some hospitals, without any such examination, provided she does her work and behaves properly. (2) Whether the ordinary course of three years in a general hospital or poor law infirmary fits nurses well for district or private work. In my opinion nurses thus trained are apt to think they must have plenty of everything to hand—in fact, are in danger of becoming extravagant. Would this be best met by another year, practically a fourth, as is in vogue in one or two hospitals, when the nurses could go out and do district work under supervision, or practise private work? (3) The few nurses who insure either in the Pension Fund or in the Poor Law Officers' Superannuation Fund. The Pension Fund studies in detail a nurse's calling; the Poor Law Officers' Superannuation Fund does not apply to nurses in the same way, for Poor Law Officials never seem to want a woman above 40 or at most 45 years of age, and yet, I believe, they do not superannuate till 65 years old. (4) Whether a general eight, or even ten, hours working day could be considered for all hospitals. Also, it would be nice to have some ideas as to how routine ward work could best be carried out, and a nurses' time table arranged.

### THE RELIGIOUS QUESTION.

"VERITAS" writes: The question of the exclusion of Roman Catholics from some of our training schools has frequently been mentioned in your columns, and in the current number I see further information on the subject from Father Power. Is it not time that definite steps were taken to settle this vexed question? Might I suggest that all registered London nurse-training schools should be asked to state definitely whether they do or do not take Roman Catholic probationers, and that the list should be published in THE HOSPITAL. By this means Roman Catholics desirous of training would know at once where to apply, matrons of sectarian training schools would be saved the trouble of interviewing impossible probationers, and the charitable public would be enabled to distribute their donations with, I think, more satisfaction to themselves and justice to all concerned than heretofore.

### THE SALARIES OF SUTHERLAND NURSES.

MISS E. A. STEVENSON, Secretary and Superintendent of the Sutherland Benefit Nursing Association, Golspie, writes: In THE HOSPITAL of December 27th, there is a note regarding "the salaries of Sutherland nurses." As this note contains statements which are incorrect and misleading, I will feel obliged if you will insert a correct statement in next issue. The nurses at present working under this Association are by other associations similar to our own termed "Rural Maternity Nurses" or "Cottage Nurses." Their training, which is a six months' one, is paid for by the Association, and including outfit, travelling expenses, examination fee, etc., costs from £35 to £40. Wages now commence at £26 and rise to £30 per annum. After reaching £30, increases are granted on the following conditions, viz.:—(1) When, after a nurse has completed six years' continuous approved service with the Association, her average number of important cases in the three years previous to each July 31st, amount to 30 or upwards a bonus of £5. (2) When, on conditions stated above, cases amount to 25, but under 30, a bonus of £4 (3) When, on conditions stated above, cases amount to 20, but under 25, a bonus of £3. When the nurses attain the age of 60, and if they have conducted themselves to the satisfaction of the Association, there is an endowment assurance of £100 awaiting them. The annual premiums to provide this are entirely paid by the Association. The nurses are insured against sickness (infectious) and accident. In regard to the work done by some of the nurses take for an example one who in a year nursed 15 cases. Out of the 365 days she worked only some 128 or 130 days. This nurse was receiving wages at the rate of £1 10s. per case. Thanks to the untiring exertions of the Duchess of Sutherland this Association has never yet been reduced to its last half-crown. Such a fact is I think rather one for congratulation than one for (to use a good Scotch word) "girnin" at.

[According to the information supplied to "Burdett's Hospitals and Charities for 1902," the salaries of the Sutherland Nurses commenced at £20 the amount named by us; and the subscriber whose complaint we mentioned evidently believed the nurses to be fully trained.—ED. THE HOSPITAL.]

### THE CIVIL HOSPITALS AND THE ARMY NURSING SERVICE RESERVE.

"A RESERVE SISTER" writes: I am indeed honoured by having so formidable a critic as Mr. Sydney Holland, and as a member of the Service he condemns I feel that I must reply. Mr. Holland says "that a standing reserve can never supply the best nurses," "that the matrons of the principal London hospitals, with one exception, were against its formation from the first," and he complains that the members of the A N S R are "self-selected volunteers." He questions whether were I matron of a hospital I would appoint as sister a woman who might be called upon to serve her country in time of war. I answer unhesitatingly yes, provided she were in all other respects a suitable person; but as I have already written I would, for the sake of my hospital, only allow a certain proportion of my nursing staff to be members of the A.N.S.R. I do not think I am "egregiously wrong" in saying that all (matrons included) agree that a reserve of nurses to supply the needs of the army in time of war is a necessity. It is not the existence of a reserve, but its control and selection, which they question. Apparently they wish the nursing staff of their hospitals to be regarded as a national reserve, and the selection of nurses for the service of the War Office to remain entirely in their own hands. They object to the Army Nursing Service Reserve as constituted by the War Office, because its members—like those of every other society—are elected on their own application. But the members were not self-selected volunteers; they were selected by the War Office and recommended by their matrons. True they volunteered. So did the civil surgeons. Can Mr. Sydney Holland really wish to deny to professional nurses in these days of enlightenment the right to volunteer their service for the use of their country, and expect them to be content to be "supplied" to the War Office by the matrons of the hospitals as are supplied to the remount department by the dealers? Mr. Sydney Holland says: "A standing reserve can never supply the best nurses." Can it be said that it has had a fair chance? On Mr. Holland's own showing, the matrons of the leading hospitals discouraged their best nurses from joining it. The War Office drew up an admirable set of rules for the admission of candidates, but the hostility of the matrons made it difficult to obtain a large enough number of nursing sisters fulfilling these conditions; therefore they had to lower the standard, and enlist "specials." Thus, it is generally considered essential that an army sister should be a getlewoman, and that the candidate should be able to speak a foreign language. But after a time the social recommendation to prove the candidate's gentle birth was no longer exacted, and I have met more than one sister who had considerable difficulty in speaking her own language, and certainly had no acquaintance with any other. Also, the three years' certificate from a general hospital was not always insisted on, and sometimes the age limit set aside. I did not merely "admit," but stated as emphatically as I could, that private nurses are not suitable as army sisters. The fact of their having become private nurses was probably because they were more suited to that work than to the management of a ward, and it is the management of a ward which is an essential in a military hospital. I stick to my guns and maintain that if the matrons would work for the Reserve, instead of against it, by encouraging their best nurses to become members in a reasonable proportion to the strength of their staff, a very high standard of nurses would be ensured, and the matrons would have practically the selection of the A.N.S.R. members as they seem to wish, while the voluntary character of the service would be preserved. The War Office would then be able to enforce its original conditions, and only fully-trained gentlewomen of unimpeachable character would be admitted members of the A.N.S.R.

### SUPERINTENDENT NURSES AND POOR LAW PENSIONS.

"A CERTIFICATED NURSE PREMIUM PAYER" writes: Respecting nurses and Poor Law pensions, kindly allow me as a M. A. B. nurse and a premium payer, both to the Poor Law Superannuation and the Royal National Pension Fund for Nurses (one of the first 1,000), a little space. I wish particularly to express my thanks, also the thanks of many other nurses with whom I am associated, to the Editor of THE HOSPITAL for the publicity given in the issue of January 10th, 1903, to Mr. Preston Thomas's candid confession in his report as inspector of Poor Law Administration. He emphasises two very important points in reference to nurses and the present conditions under which they serve, if appointed by the L. G. B. Mr. Preston Thomas has evidently grasped the nurses' position, and he put the case as to her monetary outlook and pension in a nutshell. In fact, he expresses our sentiments almost entirely, and coming from such an able authority, little need be added, only I should like to ask all whom it may concern, as well as all who have the opportunity and power, to still further drive home the urgent necessity for a State pension to such nurses after 20 or 25 years' service. Surely, the nurses in infirmaries, together with nurses in hospitals for infectious diseases, demand State consideration in the matter of pensions, no less than Army nurses.

### Wants and Workers.

I HAVE eight volumes of THE HOSPITAL, from vol. xxv. to xxxii. If any of the readers of THE HOSPITAL would like any or all of them I would forward them on application. Address S. E. W., 9 Courtney Road, Highbury, N.

### Death in Our Ranks.

WE regret to announce the death, from chronic Bright's disease, of Miss Emma Sophia Wilson, who passed away on Friday last. Miss Wilson was trained at the Workhouse Infirmary, Brownlow Hill, Liverpool, and afterwards took up district nursing in connection with the North London Association. This post she was compelled to relinquish before half her time of contract had expired. Subsequently, when her health permitted, she nursed privately, but during the last two years she has been obliged to entirely give up work.

### Appointments.

[No charge is made for announcements under this head, and we are always glad to receive, and publish, appointments. But it is essential that in all cases the school of training should be given.]

BARNET UNION.—Miss Florence Grey has been appointed assistant nurse. She was trained by the Meath Workhouse Nursing Association at St. Peter's Home, Kilburn, N W.

CITY ISOLATION HOSPITAL, NOTTINGHAM.—Miss Mary Tait has been appointed sister. She was trained at Ashton-under-Lyne District Infirmary, and was afterwards private nurse at Oldham Nursing Institution.

COLCHESTER HOSPITAL.—Miss F. M. Page has been appointed sister. She was trained at the Royal Devon and Exeter Hospital, Exeter, and has since been sister at East Dulwich Infirmay.

EASTRY UNION, SANDWICH.—Miss May McSweeney has been appointed assistant nurse. She was trained by the Meath Workhouse Nursing Association at the Children's Hospital, Temple Street, Dublin, Ireland.

HUNGERFORD UNION INFIRMARY.—Miss Nellie Peploe has been appointed assistant nurse She was trained by the Meath Workhouse Nursing Association at the Royal Hospital for Diseases of the Chest, City Road, E.C.

ISOLATION HOSPITAL, LOWESTOFT.—Miss Annie Lawton has been appointed matron. She was trained at Monsall Hospital, Manchester, and has since been charge nurse and deputy matron at the City Hospital South, Grafton Street, Liverpool.

MACCLESFIELD ISOLATION HOSPITAL.—Miss Louie Chapman has been appointed matron-nurse. She was trained at the City of Birmingham Fever Hospital, and has since been charge nurse at Crewe Isolation Hospital.

STONE JOINT HOSPITAL.—Miss Georgina McLeay has been appointed nurse-matron. She was trained at Chalmers Hospital, Banff, N.B., and has since been Queen's nurse at Hanley.

THANET ISOLATION HOSPITAL, RAMSGATE.—Miss Elizabeth Hales has been appointed charge nurse. She was trained at Mill Road Infirmary, Liverpool.

THE SANATORIUM, RAILWAY SERVANTS' ORPHANAGE, DERBY.—Miss Margaret Jane Quinn has been appointed nurse-matron. She was trained at St. George's Hospital, London, and has since been a member of the London Association of Nurses, New Bond Street.

### To Nurses.

**WE invite contributions from any of our readers, and shall be glad to pay for "Notes on News from the Nursing World," or for articles describing nursing experiences, or dealing with any nursing question from an original point of view. The minimum payment for contributions is 5s., but we welcome interesting contributions of a column, or a page, in length. It may be added that notices of appointments, entertainments, presentations, and deaths are not paid for, but that we are always glad to receive them. All rejected manuscripts are returned in due course, and all payments for manuscripts used are made as early as possible after the beginning of each quarter.**

### Echoes from the Outside World.

### The Movements of Royalty.

THE King arrived at Buckingham Palace from Sandringham on Monday afternoon. He drove in a plain clarence to Buckingham Palace, without an escort, but the public recognised his Majesty and he was repeatedly cheered. A new command has been issued with regard to the interesting ceremony of changing the guard, which has been wont to attract so many sight-seers every morning. Hitherto the guard has been mounted at St. James's Palace. For the future it will mount during the residence of either the King or Queen in London in the forecourt of Buckingham Palace. The change was made for the first time on Tuesday, when the 2nd Battalion of the Coldstream Guards took part, their band playing during the relief of sentries. When neither the King nor the Queen is in town, the guard will mount at at St. James's as heretofore.

### The New Archbishop of Canterbury.

THE Bishop of Winchester, who has been appointed Archbishop of Canterbury, is not yet 55 years of age. A native of Edinburgh, Dr. Randall Davidson was educated at Harrow and Trinity College, Oxford. During his last year at Harrow, a gunshot wound, the result of an accident, caused his life to be despaired of, and interfered, subsequently, with his academical career. In fact, the only doubt about the choice of Dr. Davidson for the Primacy is whether he is physically strong enough to bear the strain. His first and only curacy was at Dartford, in Kent, where he was as active amid an epidemic of small-pox as he was in maintaining a Bible class. Whether in the posts of domestic chaplain to Archbishop Tait, whose second daughter he married, Dean of Windsor, Bishop of Rochester, or Bishop of Winchester, he has always displayed the same combination of spiritual earnestness and practical sagacity. From the time Queen Victoria appointed him her sub-almoner in 1882 to her death, he enjoyed her Majesty's confidence, and it is well known that the King entertains a similar feeling towards him.

### Mr. Chamberlain at Johannesburg.

ON Friday a rumour gained currency on the Stock Exchange that Mr. Chamberlain had been assassinated in South Africa. Happily, the story was without foundation, and it is believed was a most discreditable attempt to influence the markets. The Colonial Secretary after attending a banquet at Pretoria, at which most of the chief residents of the town were present, received a deputation of leading burghers who presented him with an address embodying various demands and proposals, to which he responded by intimating that it would have been more becoming if, instead of making fresh requests, the burghers had shown some recognition of the efforts and concessions already made by the Government. He also said that the burghers who had gone to Europe ought to account for the public money they had carried with them. Proceeding from Pretoria to Johannesburg, Mr. Chamberlain had a splendid reception, an enormous crowd assembling at the Wanderers' Club ground to give him a welcome. An address was presented to him in a gold box on behalf of the town. In reply to this, and other addresses, the Colonial Secretary complimented Johannesburg on its loyalty, said that the Transvaal was the key to the situation, declared that he came to strengthen the hands of Lord Milner, and refused, "until he saw it," to believe that the Johannesburgers were unwilling to bear their share of the burden of the war. Mr. Chamberlain spent most of Saturday in receiving and hearing the views of different leading men, and this week he has been mainly engaged in holding converse with representative men.

### Emigration to South Africa.

THE first party of domestic servants for South Africa in connection with the new development of the Transvaal—fifty in number—has already sailed from these shores. They are being sent out by the South African Expansion Committee. Complaint having naturally been made by mistresses at home that it is hard on Great Britain to send to her Colonies the very class of women of which there is a scarcity in our own land, the secretary of the committee makes rejoinder that most of these girls would think it beneath their dignity to enter service in England, but do not mind doing so where they are unknown and under different conditions. Suitable hostels have been provided for their reception at Capetown and Johannesburg, as well as in Salisbury and Buluwayo. Each girl who goes out has to contribute £12 towards the cost of her passage. This is advanced to her if she cannot pay it herself, and she agrees to refund it £1 per month out of her first year's wages. No other stipulation is made, nor any contract enforced between employer and immigrant, wages being paid on a monthly basis. Remuneration starts from £3 a month, and on this scale are general servants, plain cooks, housemaids, nurses for young children, and nursery governesses. Superior servants get £4 a month, and well qualified governesses £5. There are a large number of applications, particularly on the part of those who have relatives in or near the new colonies, but at least 60 per cent. are rejected as unsuitable or fail to pass the doctor's examination. Should the immigrants turn out unsatisfactorily, there is a clause in the agreement they sign to meet the case; they are warned that the Women's Immigration Department have power to deport them as "undesirables" at 24 hours' notice, at their own expense.

### Imprisoned in the Snow.

ON Saturday a snowstorm of great violence raged throughout the wilds of Lochaber and in the higher parts of Western Inverness-shire. The passengers by the 9.25 A.M. express from Fort William to Glasgow, on the West Highland Railway, had an experience which they are not likely to forget. The train proceeded as far as Luibnachlach, where some enormous snow wreaths were encountered, which brought it to a standstill. It was backed for some distance and the drift charged, with the untoward result that the engine was thrown off the rails and the coaches were imprisoned in the snow. Relief measures were taken as soon as possible, but when the driver of a goods train who was sent to the assistance of the snowed-up train was within a mile of the latter his engine also got embedded in a drift. The station master at Corrour and others supplied what food they had to the unfortunate passengers, but it was not sufficient to allay the pangs of hunger during the period of detention, which lasted from between nine and ten in the morning to between seven and eight in the evening.

### An Italian Lady Doctor.

To the list of women who have lately successfully competed with men, and carried off honours, an Italian lady must be added. Five years ago Señora Bernardo Pirovano, a native of Crema, gained the degree of doctor of philosophy. Since then she has steadily gone on with her studies, with the result that she has now taken her degree in the medical faculty, and is the youngest doctor of medicine and surgery at the University of Pavia. She is going to settle down in Florence, and to practise as a physician for women and children. She is quite prepared to find many difficulties at the outset, and will have to overcome the prejudice which is felt by many Italian people against lady physicians, but she has plenty of perseverance as well as being really clever, so that her friends are hopeful that she will ultimately score a brilliant success.

### A Book and its Story.

### FATE AND A FIDDLER.*

"THE WINDING ROAD" is quite out of the track of the ordinary novel. It is written with much insight, with absolute sincerity, and a fine reserve. The interest in the two dominant characters, Jasper and Phenice, never flags, which must inevitably have been the case had their story been in less artistic hands. Some lines on the title page from a poem, "On the Road," by Arthur Symons, give the keynote to the contents:—

"The road winds onward long and white,
It curves in mazy coils, and crooks
A beckoning finger down the height:
It calls me with the voice of brooks
To thirsty travellers in the night."

It was this voice and the onward curves of the highway that drew Phenice from the shelter and routine of the home farm, with only her old uncle and his son for company, to follow the uncertain fortunes of Jasper—"mendicant? tramp? gipsy musician? gentleman-rover? with his slender figure that might have been a boy's, and his inscrutable eyes that looked as if they had seen many a strange sight and basked under many an alien sun. Perhaps they had, or perhaps he had only been to the country that lies east of the sun and west of the moon." The author is a close observer and lover of nature, and very charming descriptions of scenery are given with an artist's eye to the endless variations which light and colour produce in the most ordinary landscape. Here at the close of a February afternoon is a glimpse of Phenice, as she climbs the downs at sunset, to stand by the old beacon, and peep over the edge of the hill at the distant view of the five counties said to be visible from it. "Up here it was very still with the hush that follows the sunset; you could almost hear the sheep cropping the grass on the hillside, half a mile away. Overhead the sky was a lambent green, melting into daffodil, which deepened to red near the horizon, and in the midst of the flush stood a little clear, cold new moon. The February air was chill and dank, but Phenice was hardy and did not heed it. This was her resting time; all her tasks followed each other with hardly an interval, but now the cows were milked and she could take a few minutes' breathing space." How vividly the girl and the scene stand out in the chill silence of the early spring afterglow!

The homely background of farm makes a fitting setting for Phenice, "who still followed the household ways in which her grandmother had brought her up. The standard at Priestlands belonged to a bygone day, but it was easy to live up to. The kitchen, to people of homely habits, was a luxurious living room. . . . Round about the chimney corner stood a high-backed oaken settle with an angle, fending off all draughts, and opposite the straw beehive chair with chintz cushions in which granddad always sat of an evening. The corner cupboard with glass doors, the eight-day clock, the huge deep chest that stood in a dim corner, the sheraton chairs, the lofty oak dresser, black with age, where bits of precious china and delft stood cheek by jowl with jugs and plates of modern crockery had an air of belonging to the room." This evening, as she was coming home, Phenice had been aroused from her day dreams by the distant sound of a violin; an inherited gift for music made the sound a welcome one to her. "At first it seemed only the wind in the tents, but gradually a tune detached itself—a gay, wild melody, with a curious lilt in it, as though the player were walking The colour deepened in her cheek and a light came into her eyes; she straightened herself and drew a little breath of expectation."

Phenice had been joined by her cousin Andrew, and as the light came into her face a shadow crossed his. It had been a tacit arrangement between the farmer, his father and himself, that he should some day marry Phenice and stay on at the old homestead. If this was the understanding it was one to which Phenice was a stranger, and in which she would not have acquiesced. It was not Jasper's first visit to the farm, and his return was not welcomed by Cousin Andrew. "There's that chap again," he said; "the fellow must be daft to come out here fiddling. Why can't he stop in the town with his like?"

"He has no 'like'" said Phenice shortly.

They reached the gate leading into the home field. Her cousin had gone on leaving Phenice to close the gate—or not. She lingered with her hand upon it. "You are not going to pass us by, Mr. Jasper." He had resumed his tune and walk. "It seems so," he said. "Are you waiting for a formal invitation from Dan? You've grown wonderfully proud, I'm thinking." "Not I. . . . Hospitality is not mine to give, with never a roof over my own head; but what I have I'm free with." "You give far more than you take, if one measures by pleasure." "Isn't that," she touched the fiddle, "worth far more than supper and a bed? Do you suppose you are ever even paid?"

"Well! well! By the way, what do you think? A fellow I met the other day at Buda Pesth said he was sure I could earn fifty pounds a night if——"

"Oh, Mr. Jasper, if? If what?"

"Oh, my dear, it was far too big an 'if.' If I would sacrifice life and freedom and this fair world to roam about in; and grind, grind, and grind till all the heart was ground out of my playing. . . . And what is fifty pounds a night, or a hundred for that matter? What, after all, is money?"

"It is a good deal," said Phenice.

The grandfather, with whom Jasper was a favourite, greeted him as they came up to the house with, "Is that you? Come your ways in and give us a tune." . . . When he was settled down comfortably in the ingle nook he clasped his hands at the back of his head and nestled himself into the bend of the chimney watching Phenice as she made preparations for supper. Amongst these homely things she went about with a grace born of harmony; there was an inner correspondence between her nature and all things simple and unpretending." We must not give away the story, but Jasper's sudden return to Priestlands was the beginning of a lengthened stay. He developed rheumatic fever and Phenice found him a difficult patient, but the doctor who was called in was wise and sensible. "Follow Nature's guidance as far as you can," he said. "Does that mean that he is to do as he pleases? Sick people often have fancies for things that do them harm, what then?" "Ah! but then he has lived so close to Nature that he is something of an animal; an animal always knows what is good for it when it is sick. It is we overcivilised beings that have unwholesome fancies." Phenice wondered whether it had been the instinct of what was good for him that had brought Jasper to her arms in his hour of need. "The Winding Road" is a book to keep, to put on one side, and return to, albeit the story, though natural, is a sad one; wherever one turns a page there is something to ponder over and enjoy.

* "The Winding Road." By Elizabeth Godfrey. (John Lane. 6s.)

### For Reading to the Sick.

WAITING ON GOD.

MAN'S weakness waiting upon God
Its end can never miss,
For men on earth no work can do
More angel-like than this.

He always wins who sides with God,
To him no chance is lost;
God's will is sweetest to him when
It triumphs at his cost.

Ill that he blesses is our good,
And unblest good is ill;
And all is right that seems most wrong,
If it be His sweet Will!

When obstacles and trials seem
Like prison-walls to be,
I do the little I can do,
And leave the rest to Thee.—*Faber.*

---

He does not need to transplant us into a different field, but right where we are, with just the circumstances that surround us, He makes His sun to shine and His dew to fall upon us, and transforms the very things that were before our greatest hindrances, into the chiefest and most blessed means of our growth. . . . No difficulties in your case can baffle Him. No dwarfing of your growth in years that are past, no apparent dryness of your inward springs of life, no crookedness or deformity in any of your past development, can in the least mar the perfect work that He will accomplish, if you will only put yourselves absolutely into His hands, and let Him have His own way with you.—*H. W. S.*

---

"His rod and His staff will comfort them" by the way, and in the end each soul that perseveres in love shall hear His voice with the words of benediction, "Well done, thou good and faithful servant; thou hast been faithful over a few things, I will make thee ruler over many things; enter thou into the joy of thy Lord."—*Benson.*

---

In truth we cannot know the faithfulness of Jesus to our own selves unless we have been called to suffer for Him. It is while we bear the cross that we learn the greatness of the love wherewith He loved us.—*Benson.*

---

When meek obedience thou hast learnt,
In silence, and unknown,
Thou shalt do perfect service there,
In presence of the Throne.

The joy that would have held thy soul
Enchained by time and sense,
With Heaven's high interest shall be given,
Thy lasting recompense.

Thou shalt be changed:—a few short days
Will be enough to bring
A glory, that through heart and flesh
Shall breath immortal Spring. *Anon.*

### Notes and Queries.

**The Editor is always willing to answer in this column, without any fee, all reasonable questions, as soon as possible.**

**But the following rules must be carefully observed:—**

**1. Every communication must be accompanied by the name and address of the writer.**

**2. The question must always bear upon nursing, directly or indirectly.**

**If an answer is required by letter a fee of half-a-crown must be enclosed with the note containing the inquiry, and we cannot undertake to forward letters addressed to correspondents making inquiries. It is therefore requested that our readers will not enclose either a stamp or a stamped envelope.**

---

*Midwives Act.*

(115) Will you kindly inform me when the "Midwives Act" comes into operation, and where I can obtain a copy of the Act? Also, is it usual or right for a midwife to attend a case of cancer, dressing the wound daily?—*Enquirer.*

1. The Act comes into operation April 1. 2. You can doubtless obtain a copy from your stationer. 3. Certainly not, if she is doing midwifery work; moreover, a midwife is by no means necessarily a nurse.

*Royal National Pension Fund for Nurses.*

(116) 1. Will you kindly give me the address of the Royal National Pension Fund for Nurses? 2. Is a license necessary for a trained nurse to receive maternity or convalescent cases, or young children?—*N. E.*

1. The address of the Royal National Pension Fund for Nurses is 28 Finsbury Pavement, E.C. 2. No license is necessary, except that in the case of infants, where more than one is received, the house must be registered.

*Maternity Nursing.*

(117) Is there any reasonable objection to a district nurse, with maternity training, attending the four or five maternity cases that occur in the year under the supervision of medical men, when she does not attend fever cases, and whilst she adopts careful antiseptic precautions? —*M. B.*

This depends much on the amount of supervision. When the Midwives Act comes into full operation it will not be possible for a district nurse to attend cases habitually and for gain unless she is registered as a midwife.

*Lectureship.*

(118) Will you kindly give me information *re* the training for lectureships on hygiene.—*E. M. G.*

Apply to the National Health Society, 53 Berners Street, W.

*St. Veronica.*

(119) Will you kindly give me the address of the Secretary of the Guild of St Veronica.—*X. Y. Z.*

Miss Darnell, The Sanatorium, Clifton College.

*Quarantine.*

(120) Is there any home where nurses may go after nursing infectious cases, or would it be advisable to start one? Where would be the most suitable place? At the seaside, or in the country?—*Sussex.*

Nurses may go to the Nurses' Hostel, Francis Street, N.W., for quarantine, as well as to several private homes. We never give advice on such matters.

*Hospital Training.*

(121) Will you kindly tell me if eighteen is too young for a girl to go as probationer; and say what hospitals will take them at that age.—*Anxious.*

Eighteen is five years too young for hospital training.

---

**Standard Nursing Manuals.**

"The Nursing Profession: How and Where to Train." 2s. net; post free 2s. 4d.
"A Handbook for Nurses." (Illustrated). 5s.
"Nursing: Its Theory and Practice." (New Edition.) 3s. 6d.
"Nursing in Diseases of Throat, Nose, and Ear." 2s. 6d.
"Surgical Ward Work and Nursing." (Revised Edition.) 3s. 6d. net; post free, 3s. 10d.
"Art of Massage." (Second Edition.) 6s.
"Elementary Physiology for Nurses." 2s.
"Elementary Anatomy and Surgery for Nurses." 2s. 6d.
"Practical Handbook of Midwifery." 6s.
"Mental Nursing." 1s.
"Art of Feeding the Invalid." 1s. 6d.

**All these are published by the SCIENTIFIC PRESS, Ltd., and may be obtained through any bookseller or direct from the publisher, 28 and 29 Southampton Street, London, W.C.**

### Travel Notes.

**By Our Travelling Correspondent.**

### CXVI.—THE HEART OF FLORENCE.

I CONSIDER this to be the Piazza della Signoria and the grand old Palazzo Vecchio, rearing its beautiful bell tower high over the heads of the worthy Florentines hurrying about their daily occupations, or adorning an elegant leisure in the vicinity of the Loggia de Lanzi, so called from the Swiss guard of Cosimo I. The majority of the men wear large brown cloaks which (flung over one shoulder, with a grace we northerners cannot acquire) disclose a green lining, dear to the artistic eye. It has ever been an unsolved problem to me, what these graceful and statuesque beings condescend to do for a living. Certainly Italians live upon very little, but some sustenance is necessary to keep body and soul together, and how do they earn it.

Lovely groups of children are observable here too, flitting in and out among the trams and omnibuses, scraping up grain that has fallen from the horses' nosebags to take home for pet pigeons—these cherubs, very noisy and of an inquisitiveness really colossal, are often models of beauty—eyes of a deep soft brown like the body of a bumble bee, skins of olive, with a touch of deep carmine in the cheeks, and clothes (often ragged) of harmonious colours, form pictures which delight the eye. Strangely enough though, Italian children are often inclined to be rude and perfect pests if you want to sketch; this is inexplicable, because no one can be more courteous, more charmingly simple or more completely nature's gentleman than an adult Italian.

### In the Footsteps of Cellini.

There are several gems in the Loggia, but with most of us the "Perseus" holds the first place. The artistic roysterer who planned and executed that marvellous work must have had a moment of supreme happiness when he saw the work which had cost him so much, at last occupying its proud position; a position unaltered for more than three hundred years. His life reads as a romance and is well worth study: those who have not time for his somewhat diffuse autobiography will find a short notice of his life in THE HOSPITAL for March 1st, where I gave a condensed account of the casting of the Perseus. Close by this masterpiece is Donatello's "Judith and Holofernes," and the "Rape of the Sabines," by Giovanni da Bologna.

I was never weary of sitting there and watching the pulse of Florentine life beating in its centre. What scenes have been enacted there; what grandeur and what horror! The end of the Pazzi conspiracy, when the Archbishop of Pisa and Francesco Pazzi were hung from a topmost window in the Palazzo Vecchio and then dashed on to the stones below; then again the mighty Savonarola, who here met his death with patience and courage—but of him I must speak later. Inside the old palace there is much of interest and of beauty. Entering you have on the right Bandinelli's "Hercules and Cacus," which drew forth such bitter scorn from Cellini; inside is a charming small court with exquisite pillars and a fountain of Verocchio, with a graceful figure of a boy and a dolphin. You will probably visit all the rooms, and especially the Chapel of S. Bernarda. Here Savonarola and his companions received the Sacrament on the morning of their execution. Savonarola himself was imprisoned in the tower, and on the night preceding he was unable to sleep on account of the suffering caused by the torture which had been applied. Nevertheless, he asked and obtained permission to celebrate mass for himself and fellow-prisoners.

### The Ringhiera.

All that is left of this raised platform now is the part in front of the main entrance to the Palazzo. It was on the Ringhiera as it existed in those days that the Papal Commisioners had to witness the degradation and execution of the great Frate. In front of this elevated pathway the pyramids of "vanities" were collected and burnt by his enthusiastic followers, in which fanatical achievements many gems of art of all kinds perished. There is an excellent account of these scenes in "Romola." Savonarola had the agony of seeing his two devoted followers executed before him—the gentle, somewhat nervous, Fra Silvestro and the indomitable Domenico.

### Dante's Neighbourhood.

Not far from the busy Piazza della Signoria in the Via S. Martino, is Dante's home, but it has been restored out of all semblance of itself. Many years after the poet's death, great men in the artistic world, such as Michelangelo, Cellini, Albertinelli, etc. . . . came to it when in the course of time it had become a noted wine shop. The Alighieri family owned several houses close round, so that it is not quite certain which of them was really the poet's birthplace. Most certainly he was married in the Church of San Martino to Gemma, in spite of his Idyll with Beatrice. Howells complains in his usual chatty strain of the offensive newness of the restoration worked upon the old homes. "I do not complain," he says, "that the restoration is bad, it is even very good, but the unrestored back is better, and I have a general feeling that the past ought to be allowed to tumble down in peace, though I have no doubt that whenever that happened I should be one of the first to cry out against the barbarous indifference that suffered it!"

He then visits the church, much delighted with the old-world arrangement for summoning the custodian. "My pull at the bell shot the sacristan's head out of the fourth story window in the old way that always delighted me, and I perceived even at that distance that he was a man perpetually fired with zeal for his church by the curiosity of strangers. I could certainly see the church—yes—he would come down and open it from the inside, if I would do him the grace to close his own door from the outside!"

There are some interesting paintings in the church relating to the works of mercy performed by S. Antonio and his followers.

---

RULES IN REGARD TO CORRESPONDENCE FOR THIS SECTION.—All questioners must use a pseudonym for publication, but the communication must also bear the writer's own name and address as well, which will be regarded as confidential. All such communications to be addressed "Travel Editor, 'Nursing Section of *The Hospital*,' 28 Southampton Street, Strand." No charge will be made for inserting and answering questions in the inquiry column, and all will be answered in rotation as space permits. If an answer by letter is required, a stamped and addressed envelope must be enclosed, together with 2s. 6d., which fee will be devoted to the objects of "The Hospital" Convalescent Fund. Any inquiries reaching the office after Monday cannot be answered in "The Hospital" of the current week.

### Where to Go.

TUESDAY, FEBRUARY 10, 10 P.M.—Grand Advertisement and Poudre Ball at the Empress Rooms, Royal Palace Hotel, Kensington, promoted by the Ladies' Association in aid of the Metropolitan Hospital.

